# Supplementary material for: A genome-wide association study identifies 5 loci associated with frozen shoulder and implicates diabetes as a causal risk factor
Source: PLoS Genet. 2021 Jun 10;17(6):e1009577. doi: 10.1371/journal.pgen.1009577 (PMC8191964; doi:10.1371/journal.pgen.1009577)
Supplement: S1 Table — A table showing results from a two-stage regression where the GRS for the exposure was regressed against the exposure, and the genetically predicted exposure was used to predict frozen shoulder. Adjusted refers to a sensitivity analysis in which both stages of the regression were adjusted for the significant variables in Table 1. (DOCX) [file pgen.1009577.s001.docx]

## Supplementary Table 1 – One Sample Mendelian Randomisation Results

| Exposure | Outcome | OR | P value |
| --- | --- | --- | --- |
| T1D-GRS | FS_ICD-10+OPCS | 1.05 (1.02-1.09) | 0.002 |
| T1D-GRS | FS_all | 1.04 (1.02-1.06) | 2x10^-6^ |
| T2D GRS | FS_ICD-10+OPCS | 1.10 (0.99-1.22) | 0.07 |
| T2D GRS | FS_all | 1.07 (1.02-1.13) | 0.006 |
| T1D-GRS (adjusted) | FS_ICD-10+OPCS | 1.05 (1.02-1.09) | 0.002 |
| T1D-GRS (adjusted) | FS_all | 1.04 (1.02-1.06) | 8x10^-7^ |
| T2D GRS (adjusted) | FS_ICD-10+OPCS | 1.08 (0.99-1.19) | 0.10 |
| T2D GRS (adjusted) | FS_all | 1.06 (1.02-1.11) | 0.007 |

Results from one sample Mendelian Randomisation analysis using two stage least squares regression
