# Supplementary material for: A genome-wide association study identifies 5 loci associated with frozen shoulder and implicates diabetes as a causal risk factor
Source: PLoS Genet. 2021 Jun 10;17(6):e1009577. doi: 10.1371/journal.pgen.1009577 (PMC8191964; doi:10.1371/journal.pgen.1009577)
Supplement: S2 Table — A table showing results the IVW analysis. Meta Analysis 1 refers to using the betas and standard errors from the meta-analysis GWAS with FinnGen using ICD10 + OPCS from UKBB. Meta Analysis 2 refers to the same using ICD10 + OPCS + GP records from UKBB. The P het column contains heterogeneity. (DOCX) [file pgen.1009577.s002.docx]

## Supplementary Table 2 – IVW Mendelian Randomisation Results

| Exposure | Outcome | OR | P value | P het |
| --- | --- | --- | --- | --- |
| T1D | UKBB ICD-10 | 1.05 (1.03-1.07) | 7e-5 | 0.39 |
| T1D | UKBB GP | 1.03 (1.02-1.05) | 3e-6 | 0.32 |
| T1D no DR3/DR4 haplotyping | UKBB ICD-10 | 1.04 (1.01-1.06) | 0.002 | 0.42 |
| T1D no DR3/DR4 haplotyping | UKBB GP | 1.03 (1.02-1.04) | 6e-5 | 0.23 |
| T1D no DR3/DR4 haplotyping | FinnGen | 1.05 (1.02-1.08) | 8e-4 | 0.55 |
| T1D no DR3/DR4 haplotyping | Meta Analysis 1 | 1.04 (1.02-1.06) | 2e-4 | 0.05 |
| T1D no DR3/DR4 haplotyping | Meta Analysis 2 | 1.03 (1.02-1.04) | 2e-5 | 0.08 |
| T1D no HLA | UKBB ICD-10 | 1.03 (0.97-1.10) | 0.28 | 0.39 |
| T1D no HLA | UKBB GP | 1.03 (0.99-1.06) | 0.14 | 0.12 |
| T1D no HLA | FinnGen | 1.05 (0.98-1.12) | 0.16 | 0.44 |
| T1D no HLA | Meta Analysis 1 | 1.04 (0.99-1.10) | 0.15 | 0.06 |
| T1D no HLA | Meta Analysis 2 | 1.03 (1.00-1.06) | 0.09 | 0.04 |
| T2D | UKBB ICD-10 | 1.07 (0.98-1.16) | 0.14 | 0.24 |
| T2D | UKBB GP | 1.04 (1.00-1.09) | 0.06 | 0.03 |
| T2D | FinnGen | 1.00 (0.92-1.09) | 0.92 | 0.75 |
| T2D | Meta Analysis 1 | 1.04 (0.97-1.11) | 0.26 | 0.09 |
| T2D | Meta Analysis 2 | 1.04 (0.99-1.08) | 0.09 | 0.01 |

Meta Analysis 1 refers to using the betas and standard errors from the meta-analysis GWAS with FinnGen using ICD10 + OPCS from UKBB. Meta Analysis 2 refers to the same using ICD10 + OPCS + GP records from UKBB. The P het column contains heterogeneity statistics
