# Supplementary material for: A genome-wide association study identifies 5 loci associated with frozen shoulder and implicates diabetes as a causal risk factor
Source: PLoS Genet. 2021 Jun 10;17(6):e1009577. doi: 10.1371/journal.pgen.1009577 (PMC8191964; doi:10.1371/journal.pgen.1009577)
Supplement: S3 Table — A table showing results the MR-Egger results. Meta Analysis 1 refers to using the betas and standard errors from the meta-analysis GWAS with FinnGen using ICD10 + OPCS from UKBB. Meta Analysis 2 refers to the same using ICD10 + OPCS + GP records from UKBB. (DOCX) [file pgen.1009577.s003.docx]

## Supplementary Table 3 – MR-Egger Results

| Exposure | Outcome | OR | P value | p int |
| --- | --- | --- | --- | --- |
| T1D | UKBB ICD-10 | 1.05 (1.03-1.07) | 1e-4 | 0.43 |
| T1D | UKBB GP | 1.03 (1.02-1.05) | 2e-6 | 0.11 |
| T1D no DR3/DR4 haplotyping | UKBB ICD-10 | 1.04 (1.02-1.07) | 0.004 | 0.66 |
| T1D no DR3/DR4 haplotyping | UKBB GP | 1.03 (1.02-1.05) | 5e-5 | 0.16 |
| T1D no DR3/DR4 haplotyping | FinnGen | 1.05 (1.02-1.09) | 0.003 | 0.95 |
| T1D no DR3/DR4 haplotyping | Meta Analysis 1 | 1.05 (1.02-1.07) | 0.001 | 0.78 |
| T1D no DR3/DR4 haplotyping | Meta Analysis 2 | 1.03 (1.02-1.05) | 3e-5 | 0.25 |
| T1D no HLA | UKBB ICD-10 | 1.07 (0.96-1.19) | 0.21 | 0.43 |
| T1D no HLA | UKBB GP | 1.08 (1.02-1.14) | 0.009 | 0.03 |
| T1D no HLA | FinnGen | 1.06 (0.94-1.19) | 0.35 | 0.87 |
| T1D no HLA | Meta Analysis 1 | 1.06 (0.97-1.17) | 0.20 | 0.57 |
| T1D no HLA | Meta Analysis 2 | 1.08 (1.02-1.14) | 0.01 | 0.06 |
| T2D | UKBB ICD-10 | 1.03 (0.87-1.20) | 0.75 | 0.59 |
| T2D | UKBB GP | 1.00 (0.92-1.09) | 0.96 | 0.27 |
| T2D | FinnGen | 1.06 (0.91-1.25) | 0.45 | 0.38 |
| T2D | Meta Analysis 1 | 1.05 (0.93-1.18) | 0.47 | 0.87 |
| T2D | Meta Analysis 2 | 1.01 (0.93-1.10) | 0.75 | 0.51 |

Meta Analysis 1 refers to using the betas and standard errors from the meta-analysis GWAS with FinnGen using ICD10 + OPCS from UKBB. Meta Analysis 2 refers to the same using ICD10 + OPCS + GP records from UKBB.
