# Supplementary figures and images for: A genome-wide association study identifies 5 loci associated with frozen shoulder and implicates diabetes as a causal risk factor
Source: PLoS Genet. 2021 Jun 10;17(6):e1009577. doi: 10.1371/journal.pgen.1009577 (PMC8191964; doi:10.1371/journal.pgen.1009577)

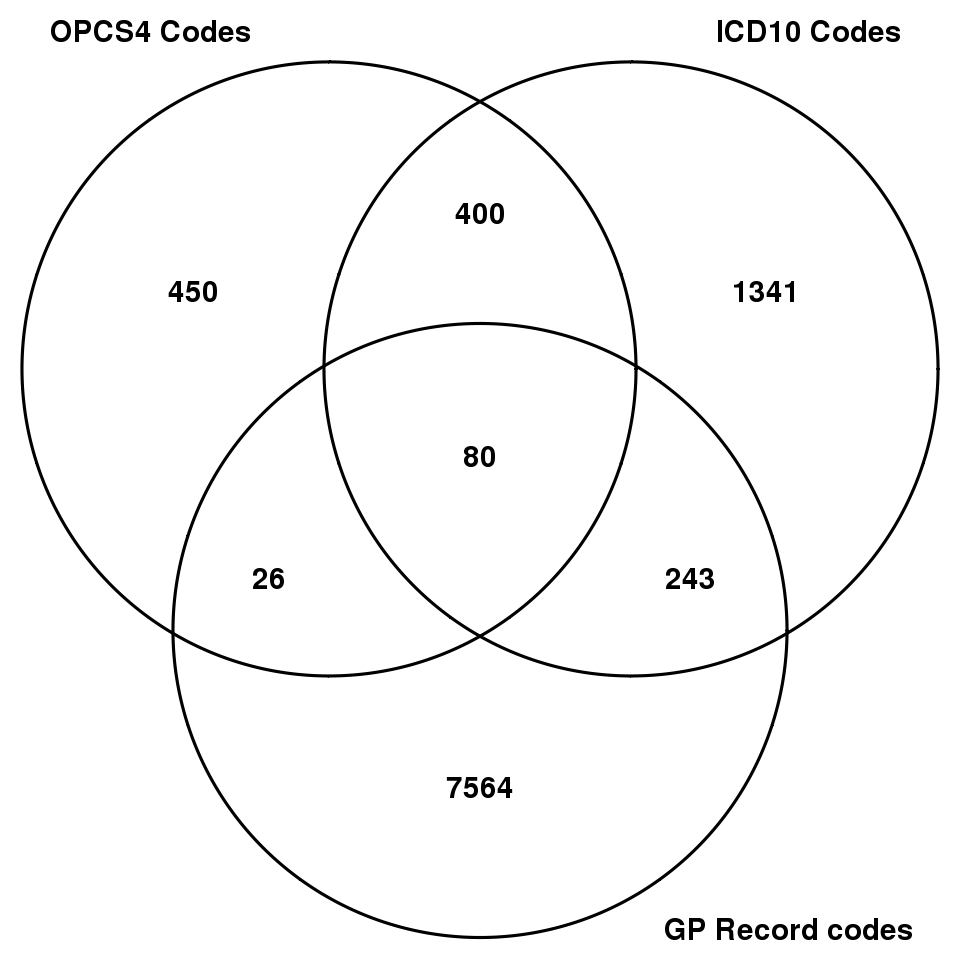

Supplement: S1 Fig — This Venn diagram shows overlap between the different case definitions of frozen shoulder (ICD10, OPCS, and GP record codes) in the UK Biobank. (TIF) [file pgen.1009577.s004.tif]

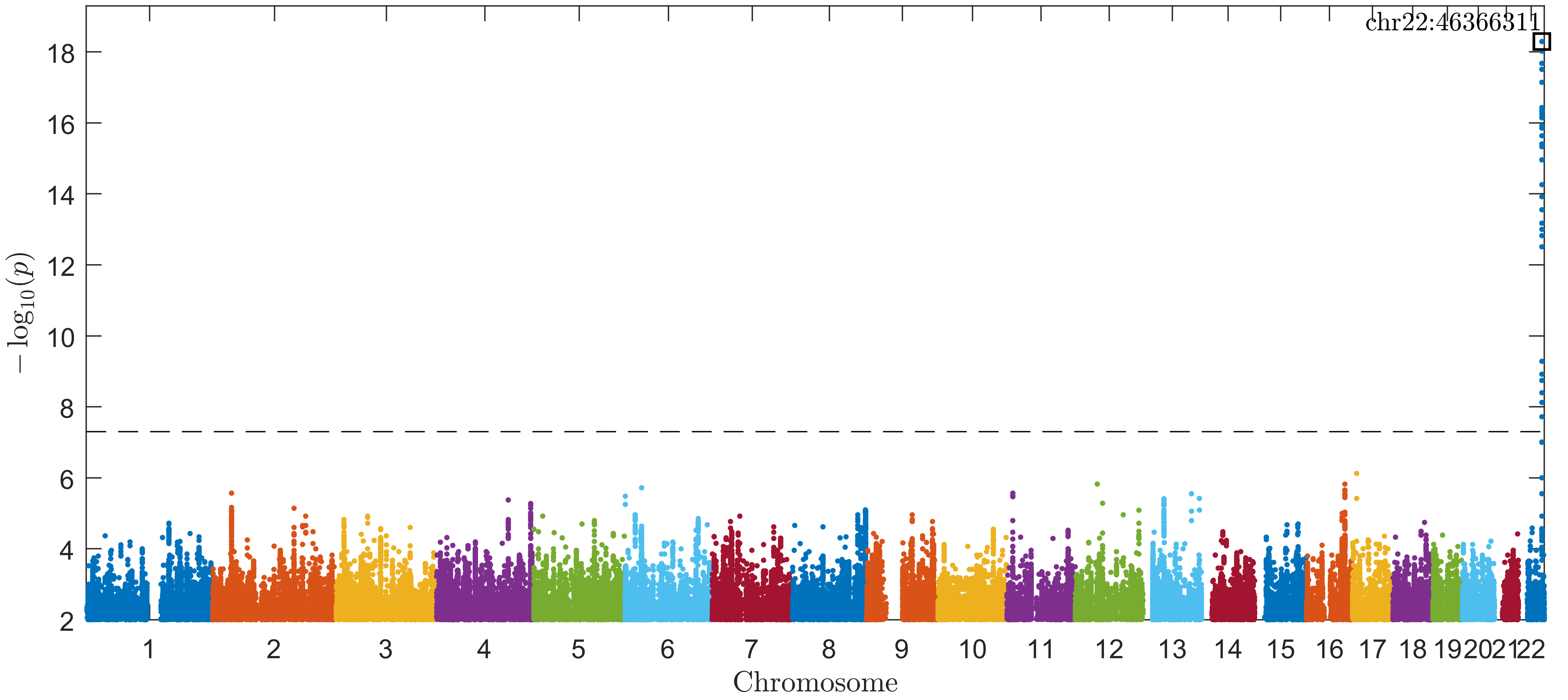

Supplement: S2 Fig — The plot shows–log10(p) values for the association of each single nucleotide polymorphism [SNP] in the HRC Imputation Panel and their association with UK Biobank frozen shoulder cases defined by ICD10 and OPCS codes. The horizontal dashed line is the genome-wide significance threshold at p = 5×10^–8^. (TIF) [file pgen.1009577.s005.tif]

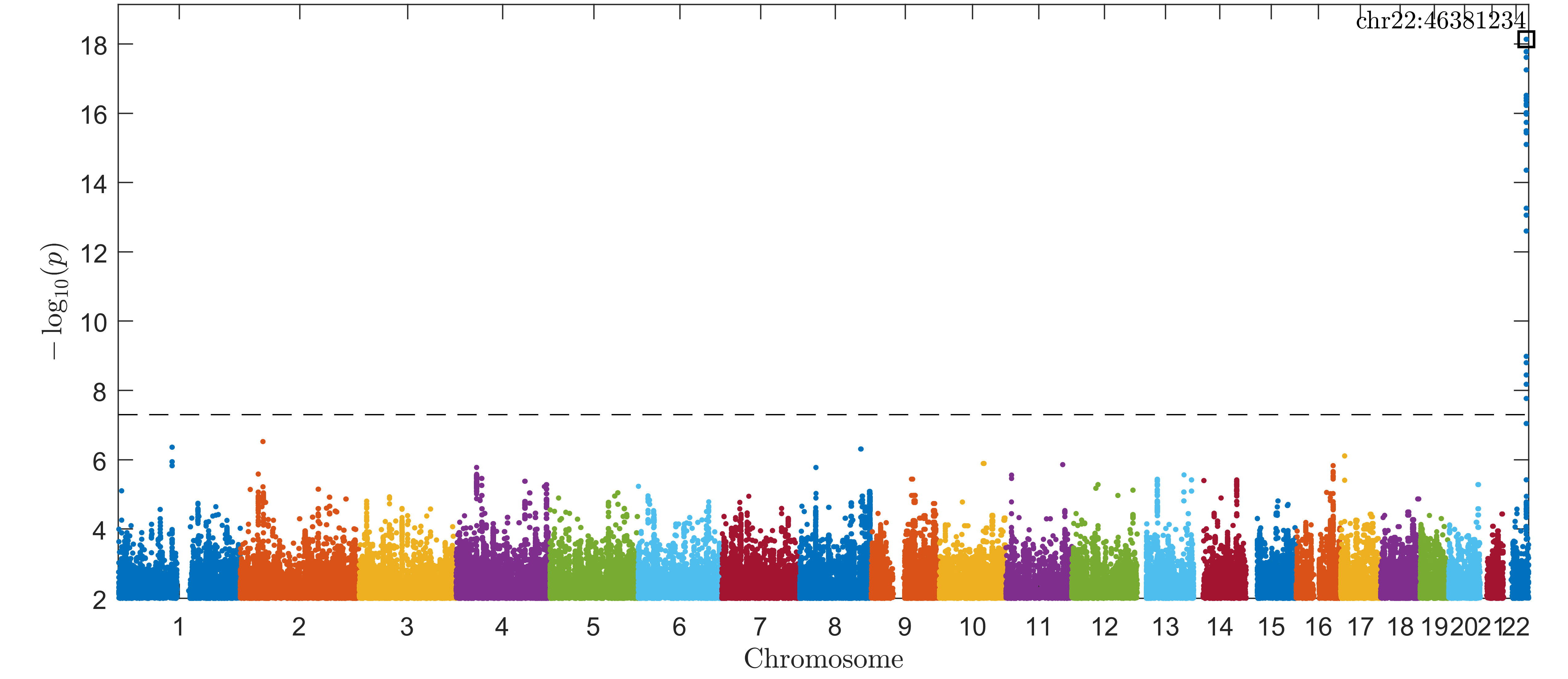

Supplement: S3 Fig — The plot shows–log10(p) values for the association of each single nucleotide polymorphism [SNP] in the HRC Imputation Panel and their association in the meta-analysis using UK Biobank ICD10 + OPCS and FinnGen. The horizontal dashed line is the genome-wide significance threshold at p = 5×10^–8^. (TIF) [file pgen.1009577.s006.tif]

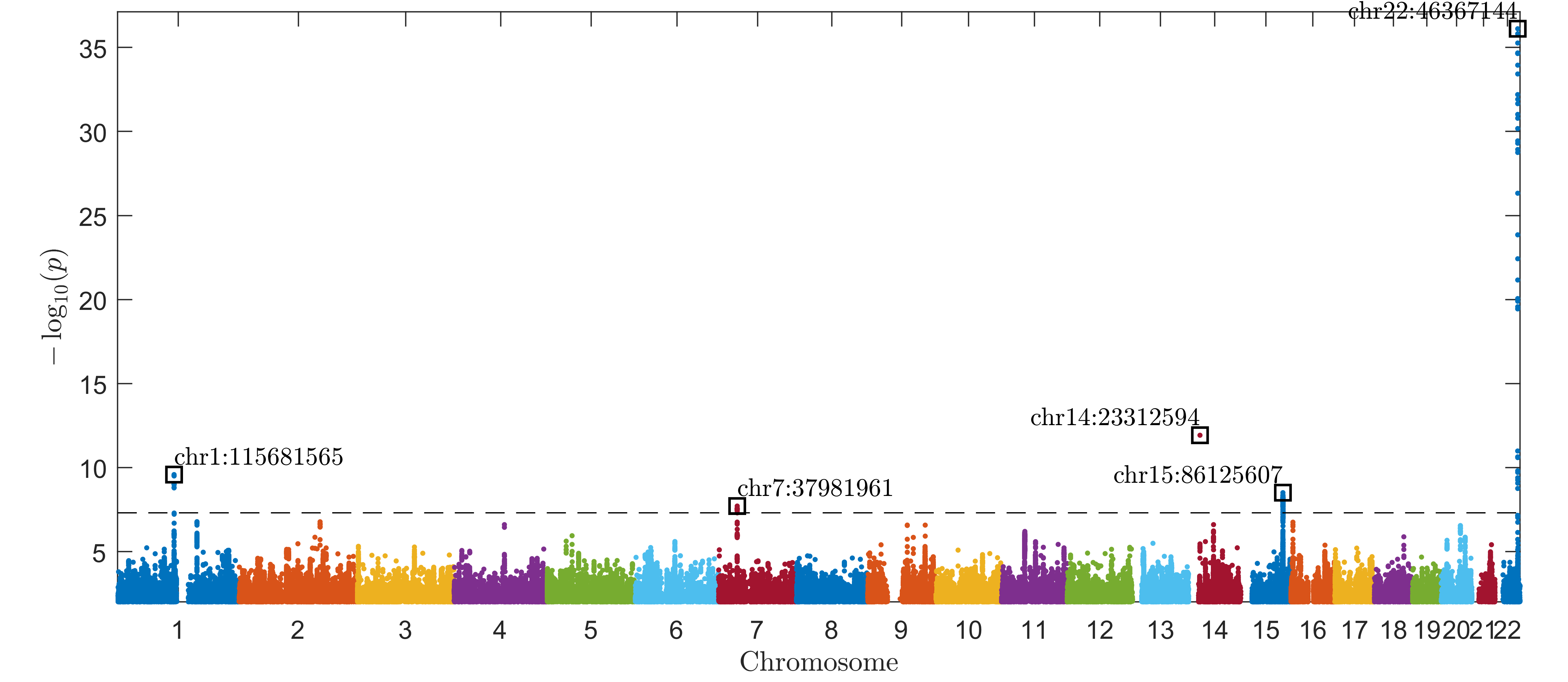

Supplement: S4 Fig — The plot shows–log10(p) values for the association of each single nucleotide polymorphism [SNP] in the HRC Imputation Panel and their association in the meta-analysis using UK Biobank ICD10 + OPCS + GP Records and FinnGen. The horizontal dashed line is the genome-wide significance threshold at p = 5×10^–8^. (TIF) [file pgen.1009577.s007.tif]

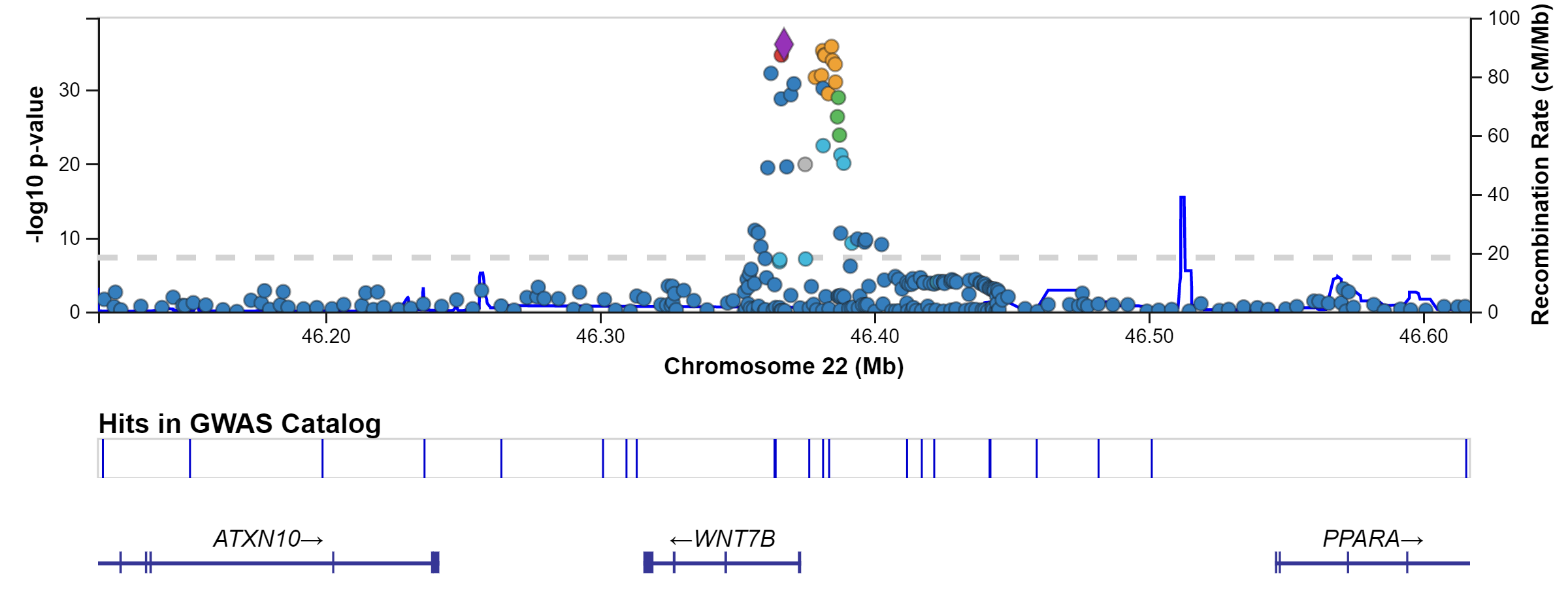

Supplement: S5 Fig — (TIF) [file pgen.1009577.s008.tif]

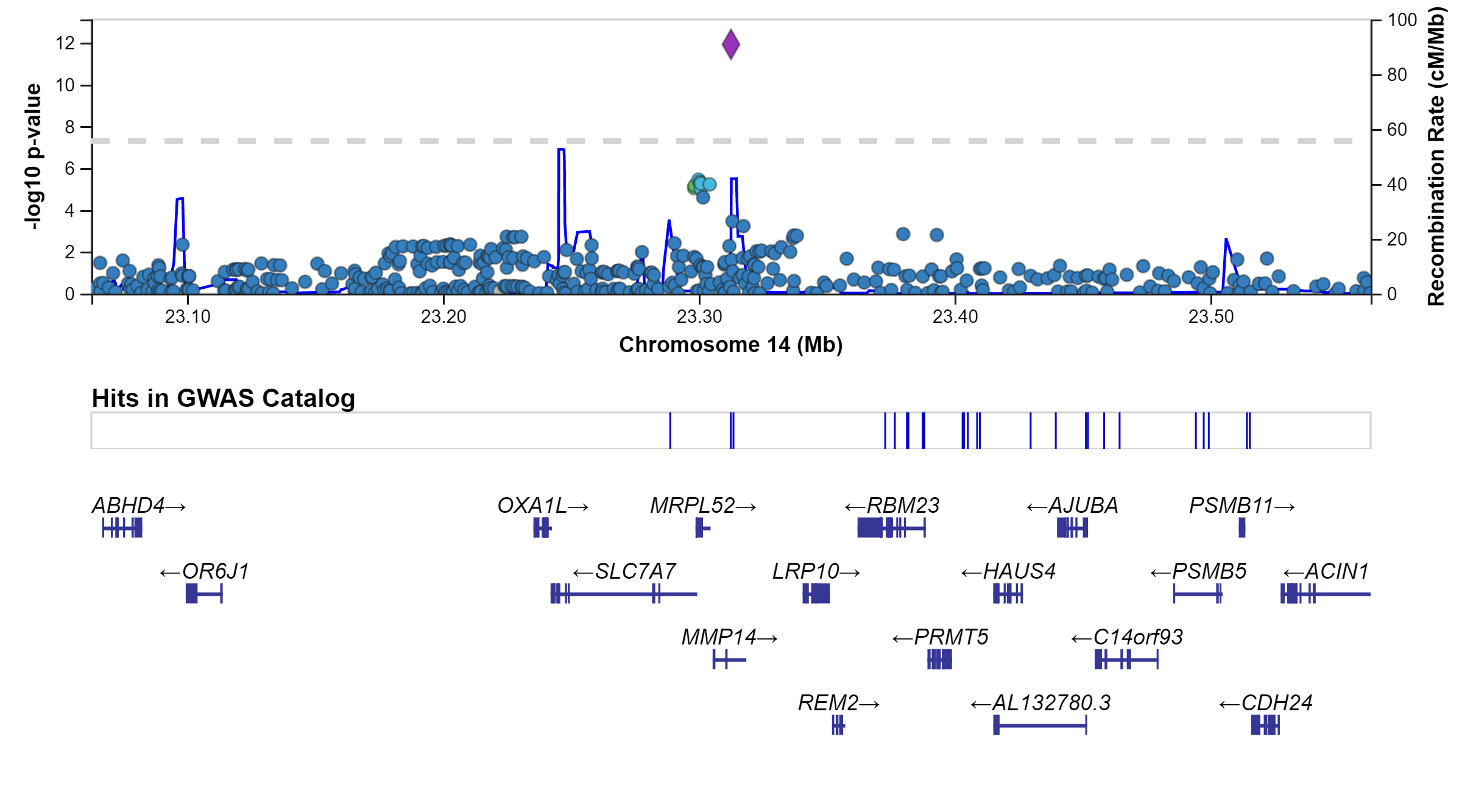

Supplement: S6 Fig — (TIF) [file pgen.1009577.s009.tif]

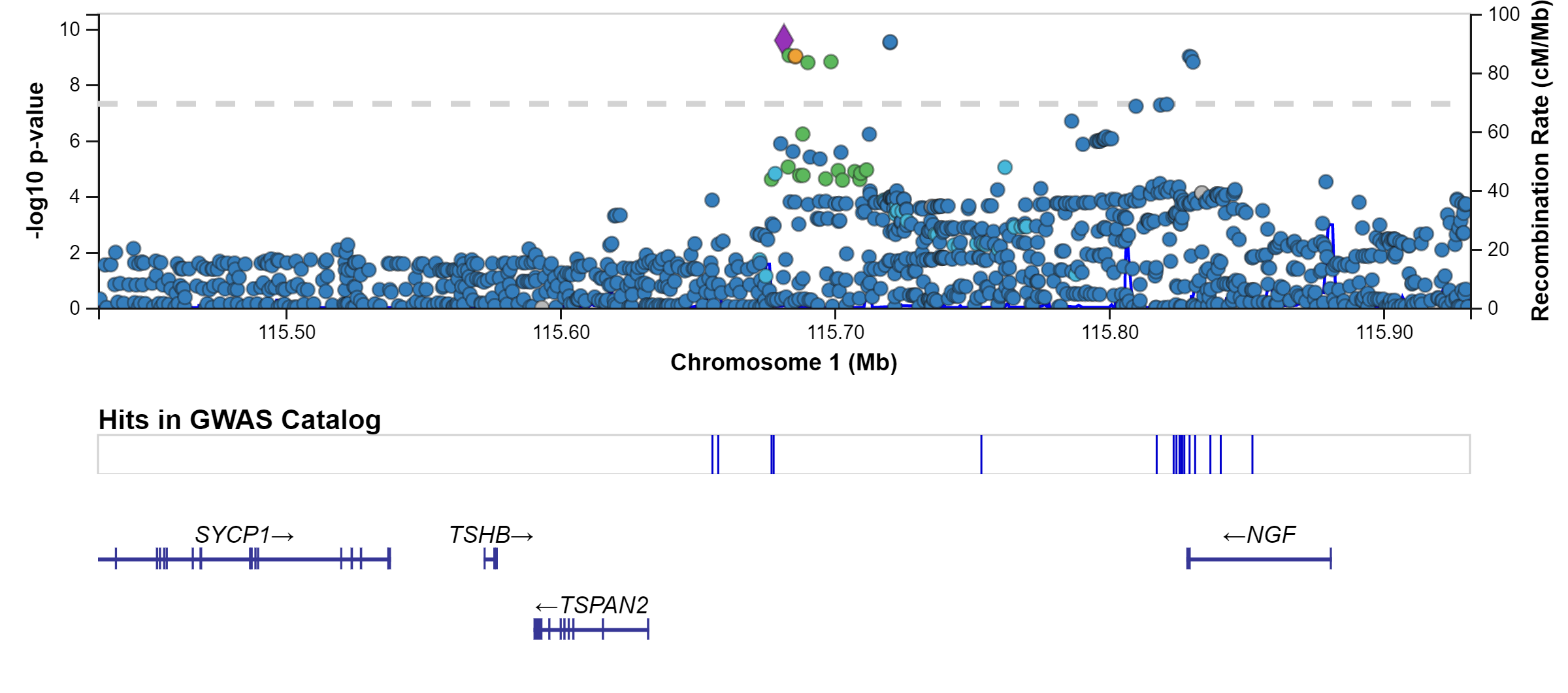

Supplement: S7 Fig — (TIF) [file pgen.1009577.s010.tif]

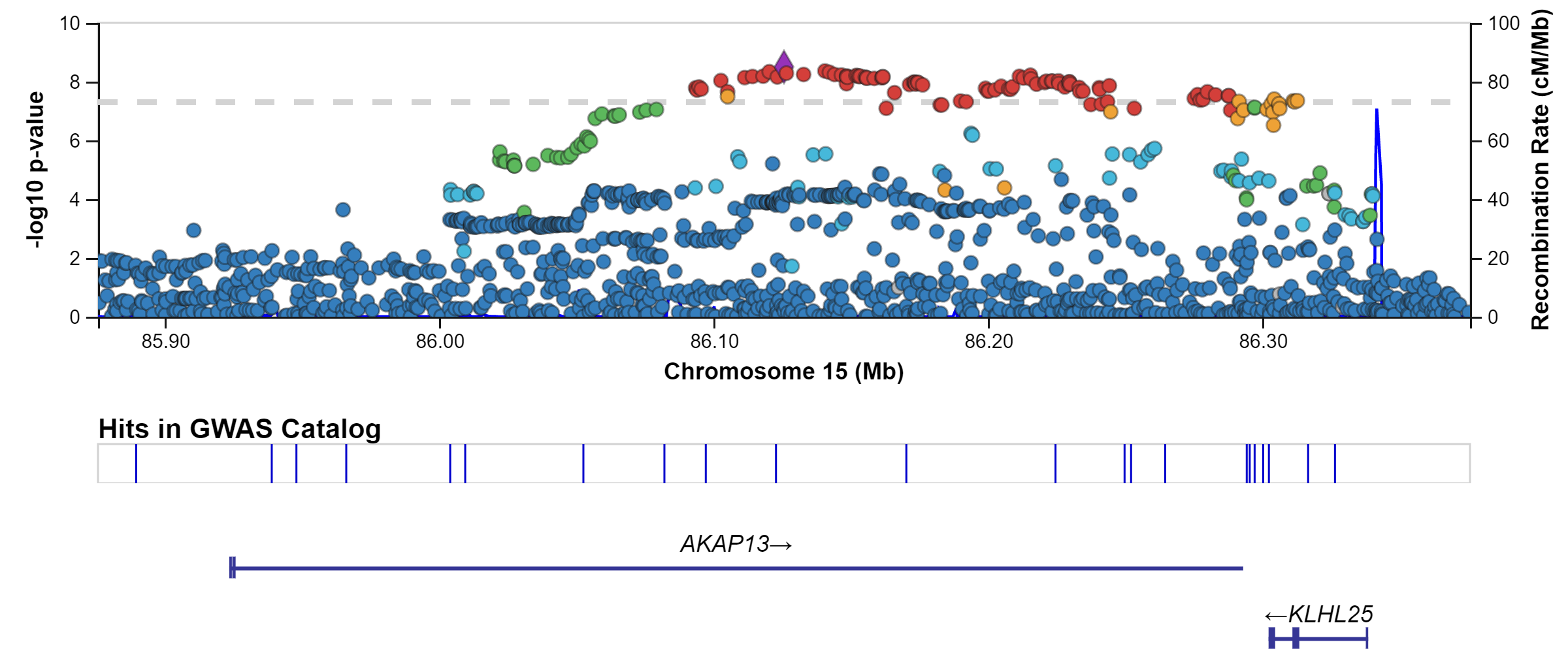

Supplement: S8 Fig — (TIF) [file pgen.1009577.s011.tif]

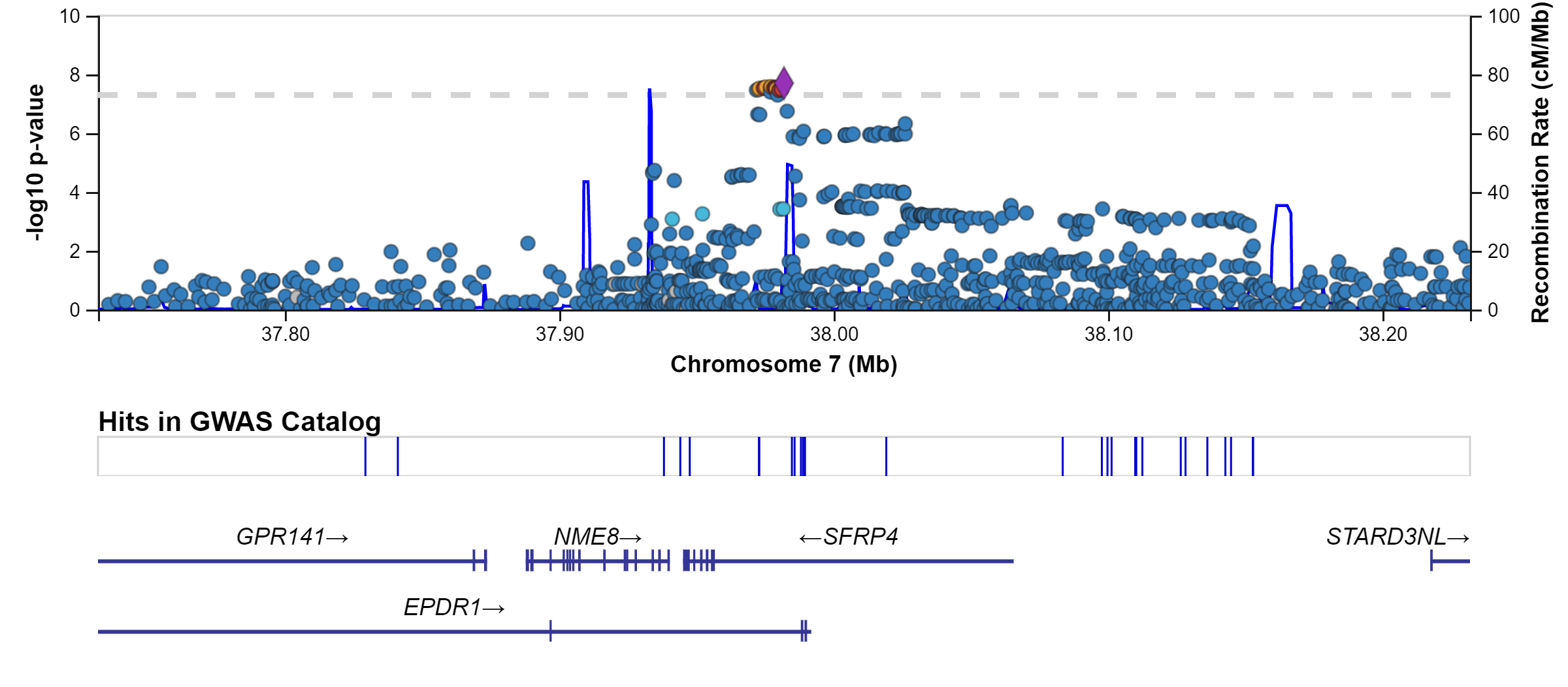

Supplement: S9 Fig — (TIF) [file pgen.1009577.s012.tif]

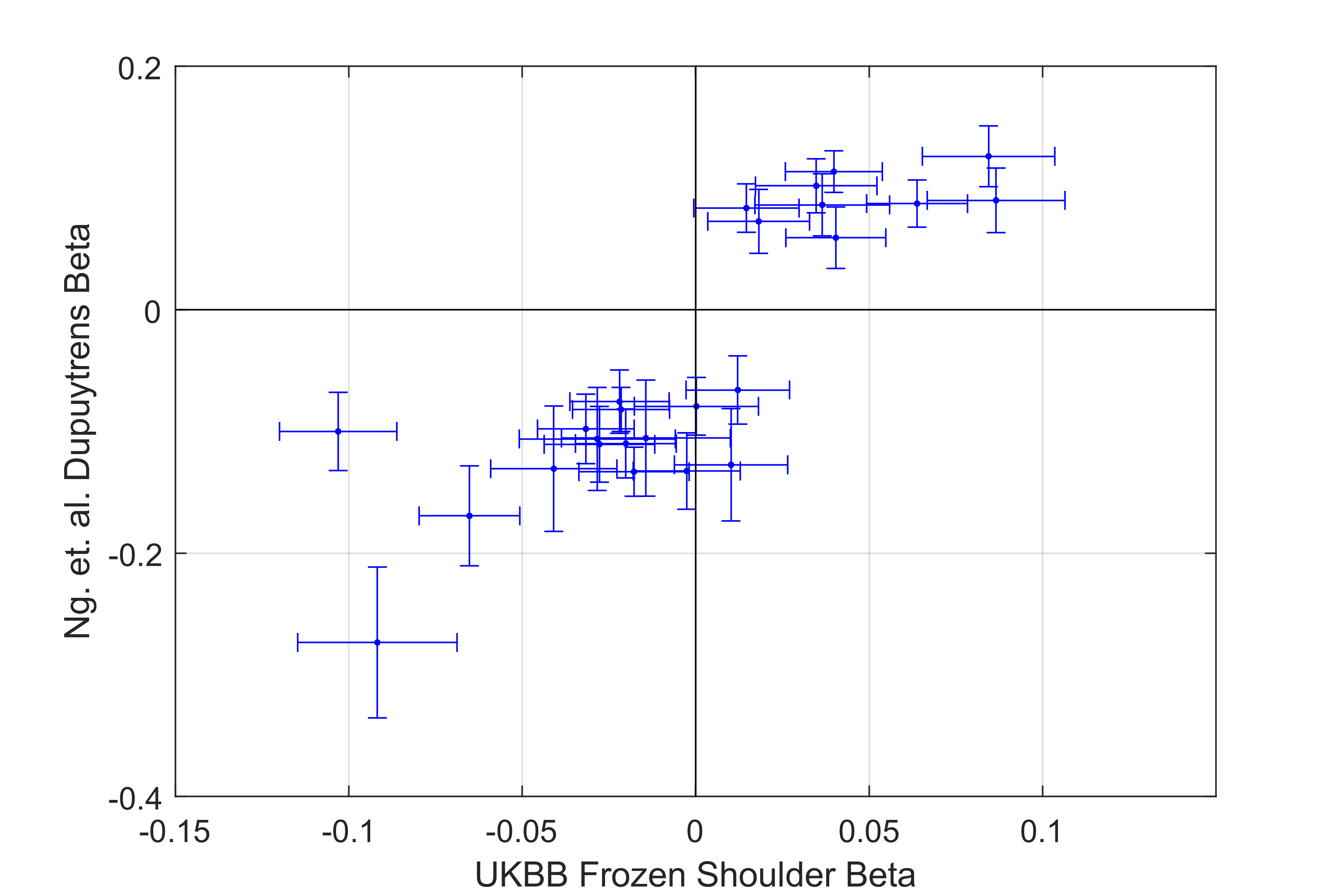

Supplement: S10 Fig — Betas and 95% CIs for association between Frozen Shoulder and between Dupuytren’s Disease for all Dupuytren’s SNPs in Ng. et. al. (TIF) [file pgen.1009577.s013.tif]
