# Supplementary material for: A genome-wide association study identifies 5 loci associated with frozen shoulder and implicates diabetes as a causal risk factor
Source: PLoS Genet. 2021 Jun 10;17(6):e1009577. doi: 10.1371/journal.pgen.1009577 (PMC8191964; doi:10.1371/journal.pgen.1009577)
Supplement: Attachment — Submitted filename: Review_Response.docx [file pgen.1009577.s014.docx]

**Response to Reviewers comments**

We thank the reviewers for their positive and constructive comments. We have responded point by point below.

**Reviewer 1:**

I enjoyed reading the paper. The work is novel, and will be of interest to the scientific community. The paper overall is well-written. I have suggested major changes that must be performed before acceptance, and would favour the authors being given a chance to perform these analyses and address my comments in a re-submission.

Major points

1. The authors have used only diagnostic codes to define disease cases for both FS and DD. This seriously underestimates the number of cases, as many are only identified by surgical (OPCS) codes. For example, for FS, a quick browse of the UK biobank data showcase reveals the following numbers of cases:

Code Cases
ICD10 – M750 2314
OPCS – W781 922

This misclassification may have led to a critical loss of power to detect association (I note several sub-threshold loci in figure 1). This is a missed opportunity, and should be rectified before publication.

Surgical codes for DD can be found in supplementary data file of <https://doi.org/10.1038/s41598-020-73595-y>

***Response:*** *We thank the reviewer for the positive comments. Our original focus on the ICD10 code defined cases was for 2 main reasons. Firstly, because our clinical co-authors advised that the GP records were likely not specific enough and secondly for consistency with the replication cohort which only used ICD10 coding. However, we agree with the reviewer that we should include all cases and then we can perform sensitivity analyses to assess the reliability of the GP record data. We have therefore repeated our GWAS using Frozen Shoulder cases from all 3 sources. The Venn Diagram below, which has been added as a supplementary figure, shows case overlap. ~75% of all cases are diagnosed purely in GP records with no OPCS or ICD10 code.*

*Performing a GWAS of all cases returned a second genome-wide significant locus on Chromosome 1, and a single significant SNP on Chromosome 14. A GWAS of ICD and OPCS codes returned the same result as the original.*

*We now report two UK Biobank GWAS results in the paper. The first is the GWAS of all cases and the second based only on OPCS and ICD codes. For completeness, we also include the GWAS of all cases in the supplementary. We also report two meta-analysed GWAS results as per comment 3.*

*With the insights gained from performing this analysis, we have repeated all observational and MR results using the ICD10 + OPCS definition of Frozen Shoulder, adding ~600 cases to the ~2000 we had before.*

*We also note that all FinnGen analyses were previously performed on a cohort of 135k participants. We now used an updated release of the FinnGen cohort with 177k participants.*

2. In a similar vein, GP data has been recently released, and the authors identify 7,913 cases of FS in the GP data. These cases are also misclassified in the GWAS analysis, and should be included as cases (note only 323 overlapped).
The authors looked only at the associated variant in this data I think, though it is not clear as there is no methodology reported for this part of the genetic analysis. The authors should combine the cases from both hospital records above and GP records into a proper case-control cohort to avoid misclassification and maximise power. They should then do a single proper GWAS. This again is a major missed opportunity to more accurately define the genetic architecture of FS, and should be performed before publication.

***Response:*** *Please see our response above.*

3. The reporting of the analysis in FinnGen is inadequate. Did the authors just test the single associated variant? What about suggestive variants that were below the genomewide significance threshold? Did they do a GWAS and then meta-analysis with the summary statistics? They should perform a full GWAS (or use the pre-prepared GWAS data) and meta-analysis as it is possible that this will reveal further associated loci, and further delineate the genetic architecture of FS. Again, a missed opportunity that should be rectified before publication.

***Response:*** *We have now performed two genome-wide meta-analyses, the first meta-analysing the ICD10+OPCS definition and FinnGen and the second with ICD10+OPCS+GP records and FinnGen. The second meta-analysis, the one with the largest sample size in the paper, yields five genome-wide significant associations:*

| Chr | BP | RSID | Meta Analysis | ICD+OPCS | ICD+OPCS+GP | FinnGen |
| --- | --- | --- | --- | --- | --- | --- |
| 22 | 46367144 | rs28971325 | 1.21 (1.25-1.18)  8E-37 | 1.32 (1.24-1.41) p=7E-17 | 1.20 (1.16-1.24) p=5E-29 | 1.30 (1.20-1.41)  p=6E-10 |
| 14 | 23312594 | rs1042704 | 1.12 (1.15-1.08)  1E-12 | 1.12 (1.05-1.20) p=8E-04 | 1.11 (1.07-1.15) p=1E-09 | 1.15 (1.07-1.24)  p=2E-04 |
| 1 | 115681565 | rs5777216 | 1.09 (1.11-1.06)  3E-10 | 0.91 (0.86-0.96) p=1E-03 | 0.92 (0.89-0.94) p=1E-09 | 0.94 (0.89-1.00)  p=6E-02 |
| 15 | 86125607 | rs17570529 | 0.92 (0.94-0.89)  3E-09 | 1.10 (1.03-1.17) p=4E-03 | 1.09 (1.05-1.12) p=1E-07 | 1.10 (1.03-1.18)  p=6E-03 |
| 7 | 37981961 | rs2472660 | 0.93 (0.95-0.90)  2E-08 | 1.06 (1.00-1.12) p=4E-02 | 1.07 (1.04-1.10) p=6E-06 | 1.13 (1.06-1.20)  p=3E-04 |

*We have included this information in the revised manuscript.*

*From the above table it can be seen that including the GP record cases the most associated variant, rs28971325, has a significantly weaker effect size in the GP records. This suggests that the GP records may include a substantial number of misclassified cases (with unrelated shoulder conditions) compared to the ICD-10 and OPCS definition. And although the other 4 associated loci have more consistent effect size across definitions, this is why we have focused the observational and MR analyses on these cases.*

4. Supplementary tables 2 and 3: I am unsure as to the precise definition of cases and controls here, and what the methodology for “meta-analysis” was (this is not described in the methods, and definitely needs to be), but I have a serious concern about performing meta-analysis when cases and (especially) controls are likely to be the same in two analyses. Specifically here in the “UK Biobank ICD” and “UK Biobank GP” cohorts I think it is likely that there is very large sample overlap. How is this dealt with in such a “meta-analysis”.
***Response:*** *The meta-analysis was performed only using UK Biobank ICD and FinnGen, which have no overlap. We have changed this methodology in the revised manuscript (described in methods - Replication and meta-analysis, page 5), and ‘meta-analysis’ now refers to using summary statistics from the genome-wide meta-analysis performed to address point 3.*


Minor points

1. Introduction Page 3 “Diabetes is the strongest known risk factor for Diabetes” – I think you mean for FS.

***Response:*** *This has been corrected in the revised manuscript.*

2. Remove “a deeply phenotyped…..from the UK” – PLOS genetics readers know the UK Biobank.

***Response:*** *This has been corrected in the revised manuscript.*

3. Results: “…had a lower Townsend Deprivation Index…” Not really. This is very marginally significant (if you were to Bonferroni correct your seven univariable tests), and is not significant in the multivariable model. Please remove.

***Response:*** *We agree with this comment as per the results in the original manuscript. However, we have changed our case definition to include OPCS codes as per the recommendation in comment 1. With this change, the p value is now 7e-4, and is significant at the Bonferroni level, so we have left the wording as it is.*

4. “Diabetic eye disease….” – how many variables related to diabetes did you test? Did you do a Bonferroni correction? This is very marginally “significant” (p=0.04) and is likely a false positive.

***Response:*** *We tested diabetes duration, HbA1c, and self-reported diabetic retinopathy, diabetic neuropathy and diabetic nephropathy, and use a Bonferroni corrected p value 0.01. With the new case definition, the retinopathy p value is 0.009, and is significant at the Bonferroni level if adjusted for the five diabetes-related variables we tested (duration, HbA1c and retinopathy, neuropathy and nephropathy: the most common diabetes complications). We agree some of these are only weak/nominal associations, but they are consistent with the associations for type 1 diabetes and provide some additional support for the effect of poorer glycaemic control in the long term being associated with increased of frozen shoulder.*

5. Locuszoom should be LocusZoom

***Response:*** *This has been corrected.*

6. Page 7 – what is the r2 between rs62228062 and rs7291412? Was rs62228062 in the 95% credible set for that locus in Ng et al? And therefore is the predisposition to FS and DD in this region of Chr. 22 likely driven by the same underlying causal variant?

***Response:*** *The r2 of the 2 variants is only 0.03 and D’ only 0.3 and rs62228062 was not in the 95% credible set in the Ng et al. paper. It’s likely that this variant was missed in the imputation panel and it is the same underlying causal variant. This is supported by the strong overlap across loci for Dupuytren’s and frozen shoulder loci.*

7. Page 7 – how many cases overlapped between FS and DD?

***Response:*** *There was limited overlap between Frozen Shoulder and Dupuytren’s disease observationally: 61 (2.4%) of cases in the ICD10+OPCS definition and 198 (2.0%) had an ICD10 code for Dupuytren’s disease. This has been added to the revised manuscript*

8. Page 7 – I think the misclassification bias discussed above extends to the MR analysis, but the methodology of which cases and controls were used in this analysis is unclear - the methods section needs to be very clearly written.

***Response:*** *We have expanded our description of how the MR analysis was performed (methods – Mendelian randomisation, page 5 and 6).*

9. Page 9 – replace elongated with extended.

***Response:*** *This has been corrected*

10. Page 9 – “…chronic condition like DD…” – please read up a bit on DD. It is a fibrotic condition just like FS, it has a similar age of onset (mean 63 years). Many patients with DD also get FS. It is not at all unexpected that both conditions might share some genetic predisposition. For example:
<https://doi.org/10.1302/0301-620X.77B5.7559688>
<https://doi.org/10.1067/mse.2001.112883>

***Response:*** *We agree and we have made this clearer throughout the manuscript and expanded the analysis showing the genetic overlap between frozen shoulder and Dupuytren’s. We have clarified that the conditions are distinct because of the often transient nature of frozen shoulder compared to Dupuytren’s disease.*

11. Page 9, final paragraph, remove the first sentence – it is repetitive, and also misleading, as only Type1 DM was really shown to be causative.

***Response:*** *This sentence has been removed in the revised manuscript.*

12. Please remove all claims to identifying the “first” variant associated with FS. This should be determined in retrospect, and such claims of primacy are not fitting of a scientific article.

***Response:*** *These changes have been made*

Tables

1. Table 1 should have numbers of participants, not just percentages. I don’t know if all of the cases in UK Biobank were included in this analysis, or just the ones identified by ICD10 coding. It should be the former – if not, please re-run the analyses.

***Response:*** *We have re-run all observational analyses using the ICD-10 + OPCS definition. We did not include the GP records, as 1) we remain concerned about the specificity of the GP records (please see our response to point 3) and 2) the study is well powered with ICD10 and OPCS definitions. We have now included the numbers of participants in this analysis, as they were performed on unrelated individuals and have a smaller sample size than the GWAS (where we used an approach that adjusts for relatedness).*

Reviewer #2: The authors conducted a GWAS on frozen shoulder in the UK Biobank sample, and replicated the finding using available GWAS results of a well-powered independent cohort. Using the GWAS results, 1-sample and 2-sample Mendelian randomization analyses were conducted revealing type 1 diabetes but not type 2 diabetes as causal risk factor for frozen shoulder. The analyses were generally sound, the findings robust, and the paper is well written. However, more details regarding the analysis methods need to be provided, and several points listed below should be addressed or clarified.

Although the conducted discovery-replication approach is valid and provides a robust result, it would be of interest to perform additionally a combined UK Biobank and FinnGen GWAS meta-analysis (without replication) to see, if there are any additional genome-wide significant associations on frozen shoulder by maximizing the sample size in the discovery stage.

***Response:*** *We have repeated the original UKBB GWAS to include OPCS codes as well as ICD-10 codes. We have also run a GWAS meta-analysis of this GWAS with the FinnGen GWAS. We see no additional genome-wide significant associations with the ICD10 + OPCS meta-analysis, but four additional associations using the GP records (recommendation of Reviewer 1). However, we have concerns over the specificity of the GP record coding. Please also see our response to Reviewer 1 comment 1 and 3.*

Methods: please provide how significance was defined in the observational analysis, the MR analysis, the GWAS, and provide the replication criteria applied for the GWAS results.

***Response:*** *P-value thresholds and have been explained throughout the paper. We now clarify in the Observational Analyses methods section how multiple testing was controlled for. 0.01 and 5e-8 were used as thresholds for MR and GWAS respectively. We now clarify we sought replication for genome-wide significant SNPs, but perform a full genome-wide meta-analysis and demonstrate consistency of effect sizes and significance across studies for all genome-wide significant SNPs in the largest meta analysis.*

How was required independence of instruments (SNPs) for the MR assessed?

***Response:*** *The SNPs in T1D-GRS are independent (r2<0.1), apart from the SNPs which are used to tag the DR3/DR4 haplotype. We have performed an analysis excluding the HLA variants and the effect sizes remain similar.*

Results page 6, first paragraph: It’s not quite clear how to “strong” associations were defined and compared, given that the effects presented in Table 1 are based on a mixture of continuous variables on different scales, and binary traits with different prevalence used as exposure. In addition, the sentence “ In a multivariable logistic regression model only the type 1 and type 2 diabetes showed strong association” is imprecise, as also sex had an OR below 1 and a very low adjusted p-value. Please clarify.

***Response:*** *This incorrect wording has been clarified in the revised manuscript.*

Observational results were provided for the duration of diabetes, but the methods are missing describing how diabetes duration (and of which type of diabetes) was assessed. This information needs to be added.

***Response:*** *We have added which variables were used to define diabetes duration in methods - Other phenotypes, page 4.*

Regarding the 1-sample MR, please provide more details how and on which scale the exposure (i.e. diabetes) was estimated using the genetic factors and included in the second stage, and explain how the causal OR on frozen shoulder can be interpreted with respect to the units of the exposure.

***Response:*** *We have included an explanation and a reference for interpreting the causal MR (methods – Mendelian randomisation, page 6, reference 26).*

As a sensitivity analysis, I suggest to adjust the first stage association in the 1-sample MR for the traits listed in Table 1 (except diabetes) to correct for potential pleiotropic effects of the SNPs that could lead to invalid instruments.

***Response:*** *New results for 1-sample MR have been provided. These use the OPCS codes as recommended by reviewer 1, and we have now included the additional covariates. We adjusted both stages of the regression and made this clear in the manuscript.*

Please provide the number of instruments included in the respective MR analyses (e.g. in the Supplementary Tables), and provide heterogeneity measures of the 2-sample MR results.

***Response:*** *These have now been included in the supplementary table with the IVW results.*

Regarding the Dupuytren’s contracture, are the two WNT7B SNPs rs7291412 and rs62228062 in linkage disequilibrium? Please provide their R².
Although WNT7B is a strong candidate gene as stated in the Discussion, I do not see the conclusion that WNT7B is the likely causal gene at the locus supported by any analysis conducted in this project – the missing eQTL associations and coding variants rather suggest the opposite. Please re-phrase or remove this conclusion.

***Response:*** *The r2 between those two SNPs was low (0.03; please also see our response to Reviewer 1, comment 6). We agree the strength of evidence for WNT7B being the causal gene is limited and we have toned this down throughout the manuscript and described it as a potential causal gene because of the known role of WNT signalling in fibrotic disease and the previous paper showing it was strongly differentially expressed in shoulder tissue from individuals with frozen shoulder.*

Please rephrase the “strong” eQTL in the Discussion because the effect size cannot be solely quantified by the association p-value. Furthermore, I encourage to re-assess the use of “strong” in combination with “OR” and “association” throughout the manuscript, and to use more appropriate wordings like higher/lower OR or association p-values.

***Response:*** *We agree and all uses of the word strong in the paper have been removed in favour of stating the p value threshold in the methods*

Discussion end of page 9: the weaker effect of type 2 diabetes on frozen might be because of earlier onset of type 1 diabetes, not because of time point of diagnosis. Please clarify or correct this statement. In the next sentence, it should be written “would have an increased risk” instead of “would be”.

***Response:*** *We agree and we have made these changes in the revised manuscript*

It should be stated were the UK Biobank frozen shoulder GWAS results will be available and accessible.

***Response:*** *We now state that all GWAS results will be made available in the GWAS catalogue*

minor issues:
- page 4: please provide the version of GTEx used
- please add the number of cases and controls to Table 1
- please provide a meaningful p-value for the “T1D no DR3/DR4 haplotyping” meta-analysis results in Supplementary Tables 2 and 3

***Response:*** *These changes have been made*

**Have all data underlying the figures and results presented in the manuscript been provided?**
Large-scale datasets should be made available via a public repository as described in the *PLOS Genetics* [data availability policy](https://eur03.safelinks.protection.outlook.com/?url=http%3A%2F%2Fjournals.plos.org%2Fplosgenetics%2Fs%2Fdata-availability&data=04%7C01%7CH.D.Green%40exeter.ac.uk%7Ce28d507d6aba408a309908d8c1590c5f%7C912a5d77fb984eeeaf321334d8f04a53%7C0%7C0%7C637471935484560571%7CUnknown%7CTWFpbGZsb3d8eyJWIjoiMC4wLjAwMDAiLCJQIjoiV2luMzIiLCJBTiI6Ik1haWwiLCJXVCI6Mn0%3D%7C1000&sdata=3779kbGLfbp6LhnrfNFW77rrhxGjrVbw%2F7jhPvaIq%2BU%3D&reserved=0), and numerical data that underlies graphs or summary statistics should be provided in spreadsheet form as supporting information.

Reviewer #1: Yes

Reviewer #2: **No:** It is not stated where the UK Biobank frozen shoulder GWAS results are available.

PLOS authors have the option to publish the peer review history of their article ([what does this mean?](https://eur03.safelinks.protection.outlook.com/?url=https%3A%2F%2Fjournals.plos.org%2Fplosgenetics%2Fs%2Feditorial-and-peer-review-process%23loc-peer-review-history&data=04%7C01%7CH.D.Green%40exeter.ac.uk%7Ce28d507d6aba408a309908d8c1590c5f%7C912a5d77fb984eeeaf321334d8f04a53%7C0%7C0%7C637471935484560571%7CUnknown%7CTWFpbGZsb3d8eyJWIjoiMC4wLjAwMDAiLCJQIjoiV2luMzIiLCJBTiI6Ik1haWwiLCJXVCI6Mn0%3D%7C1000&sdata=uJrAORiFVdtQzK27TLCoGsgdybhYJWzvZqKD12SSBkI%3D&reserved=0)). If published, this will include your full peer review and any attached files.


If you choose “no”, your identity will remain anonymous but your review may still be made public.


**Do you want your identity to be public for this peer review?** For information about this choice, including consent withdrawal, please see our [Privacy Policy](https://eur03.safelinks.protection.outlook.com/?url=https%3A%2F%2Fwww.plos.org%2Fprivacy-policy&data=04%7C01%7CH.D.Green%40exeter.ac.uk%7Ce28d507d6aba408a309908d8c1590c5f%7C912a5d77fb984eeeaf321334d8f04a53%7C0%7C0%7C637471935484570533%7CUnknown%7CTWFpbGZsb3d8eyJWIjoiMC4wLjAwMDAiLCJQIjoiV2luMzIiLCJBTiI6Ik1haWwiLCJXVCI6Mn0%3D%7C1000&sdata=LX5y98CtP9Fki3ubgnzvJYuw2zIVlLRoT%2FQB7Lizx%2B4%3D&reserved=0).

Reviewer #1: **Yes:** Dominic Furniss

Reviewer #2: No
