# Supplementary material for: A genome-wide association study identifies 5 loci associated with frozen shoulder and implicates diabetes as a causal risk factor
Source: PLoS Genet. 2021 Jun 10;17(6):e1009577. doi: 10.1371/journal.pgen.1009577 (PMC8191964; doi:10.1371/journal.pgen.1009577)
Supplement: Attachment — Submitted filename: Summary of changes.docx [file pgen.1009577.s015.docx]

Summary of changes

- Please add the effect alleles and their allele frequencies to new Table 3.

*We have added a new column with this information to Table 3*

- As far as I understand, the linear mixed model of BOLD-LMM does not generate estimates that can be directly transformed into odds ratios (compared to e.g. logistic regression). Please add to the methods how the odds ratios (and CI) were approximated from the betas and SEs obtained from the linear mixed model. Are there any known limitations of this approximation e.g. with respect to allele frequency or case-control ratio? Apologies for missing to ask this question after the initial submission already, but addressing this point would be quite important for the reproducibility and interpretation of possible uncertainties of the GWAS results.

*While BOLT-LMM does not output log-odds ratios, the instruction manual contains a formula for converting betas and standard errors to log-odds ratios. We have added the following to the manuscript: Betas and standard errors from BOLT-LMM were converted to log-odds ratios using* $log(OR)=\beta/ (\mu(1-\mu))$*, where μ is the case-control ratio, as per the BOLT-LMM user manual [16]. Standard errors were also divided by* $\mu(1-\mu)$*. This should address how log-odds ratios were obtained.*

- On page 6 (MR methods), please specify what the newly added sentence “We also performed Mendelian-Randomisation using betas and odds ratios from the two genome-wide meta-analyses.” exactly means. Did you use for the MR the betas estimated from log(odds ratio) (i.e. taking my comment on the BOLD-LMM results for FS into account)? Given that both exposures and outcome are binary traits, the simple statement “using betas and odds ratios” in the two-sample MR is somehow misleading.

*The statement ‘using betas and odds ratios’ was an error and now reads ‘using log odds ratios and standard errors’. It should now be clear that the log-odds ratios, calculated as described above, were used for the Biobank part of the MR analysis*
